# GPs’ involvement in specialised palliative home care: A mixed methods study in Germany

**DOI:** 10.1080/13814788.2022.2139824

**Published:** 2022-11-11

**Authors:** Sophie Peter, Anna Maria Volkert, Lukas Radbruch, Roman Rolke, Raymond Voltz, Holger Pfaff, Nadine Scholten, on behalf of the APVEL-consortium

**Affiliations:** a University of Cologne, Faculty of Human Sciences & Faculty of Medicine and University Hospital Cologne, Institute of Medical Sociology, Health Services Research, and Rehabilitation Science, Cologne, Germany; b Department of Palliative Medicine, University Hospital Bonn, Bonn, Germany; c Department of Palliative Medicine, Medical Faculty RWTH Aachen University, Aachen, Germany; d Department of Palliative Medicine, CIO Aachen Bonn Cologne Düsseldorf, University of Cologne, Faculty of Medicine and University Hospital Cologne, Cologne, Germany

**Keywords:** Palliative and terminal care, specialised palliative home care, home-based palliative care, general practitioners, cooperation, mixed methods design

## Abstract

**Background:**

General practitioners (GPs) are important providers of palliative home care (PHC). To deliver adequate palliative care, cooperation with specialised PHC teams is necessary. Specialised PHC is a type of care for severely ill patients by specialised providers. Little is known about the involvement of German GPs in specialised PHC.

**Objectives:**

To analyse GPs’ experience with realised and desired involvement in specialised PHC. Realised involvement means GPs took part in specialised PHC patients’ care. Desired involvement is GPs’ hoped-for cooperation with specialised PHC teams: GPs could state whether they want to stay involved, be informed, or provide medical services themselves after referral to specialised PHC.

**Methods:**

Mixed methods design (focus group with 6 GPs; survey of 445 GPs in North Rhine, Germany, about their experiences in PHC/specialised PHC): Qualitative data was interpreted using content analysis. The authors developed a questionnaire and performed descriptive analysis based on qualitative results.

**Results:**

GPs are mostly satisfied with specialised PHC teams’ care, although they report cooperation is not always optimal. GPs describe a high satisfaction with quality of care by specialised PHC teams. However, physicians with higher PC knowledge are less satisfied with specialised PHC. Also, GPs are often less involved in specialised PHC than they wish, especially when they have a higher PC qualification.

**Conclusion:**

In general, GPs are satisfied with the quality of care provided by specialised PHC teams but GPs do not always perceive cooperation as optimal. Involvement of GPs in specialised PHC needs to be improved.

KEY MESSAGES

- GPs’ satisfaction with specialised palliative home care (PHC) is generally high, but cooperation is not always perceived as optimal by GPs
- GPs with higher palliative care (PC) knowledge are less satisfied with specialised PHC
- GPs are often less involved in specialised PHC than they would like to be, especially when they have a higher PC qualification
- Involvement of GPs in specialised PHC needs improvement

## Introduction

Due to the growing number of palliative patients who (wish to) die at home [1], palliative home care (PHC) is gaining importance in primary health care worldwide [2]. Especially general practitioners (GPs) are essential PHC providers and play a central role in palliative care (PC) [2–4]: About 50% of patients with PC needs are identified in primary health care [5]. GPs mostly perform basic or general PHC, including e.g. symptom control, pain relief, psychosocial care, coordination of care and referrals to PC specialists [3,6]. If the care of palliative patients becomes too complex for GPs, it is common in the European context to refer to specialised teams. Good cooperation between GPs and specialised PHC can improve continuity of care [5]. GPs’ and specialists’ PHC can cooperate and support each other [7]. However, also competence conflicts may occur [8].

In Germany, specialised PHC is provided by PC specialist teams, including highly qualified PC physicians, nurses and other health care professionals [9]. Specialised PHC complements general PHC by GPs and is indicated for approximately 10 percent of the PHC patients with high care needs [10]. Patients are entitled to specialised PHC. It is covered by German statutory health insurance if the medical necessity and a physician’s prescription are given [5,11]. The average time for specialised PHC is 61.4 days (Median: 24 days) [11]. Most referrals are made by primary care physicians [12]. GPs report an unclear definition of their own role once specialised PHC is involved [13]. Referring patients to PC specialists may be perceived as a loss [14]. In an European comparison, Germany reports the highest number of specialised PC services; 31 percent are home PC teams [5]. However, PC provision is quite heterogeneous [13]. There is no national standardised strategy. Collaboration and coordination between GPs’ and specialists’ PHC tasks are not well defined in the German healthcare system [15].

This article’s research question is: How do GPs in North Rhine, Germany experience realised and desired involvement in specialised PHC?

## Methods

Methods and results are reported based on ‘Good Reporting of a Mixed Methods Study’ [16] (Appendix 1).

### Study design

The data analysis presented here is based on the publicly funded APVEL study (Evaluation of specialised PHC in North Rhine; fund number: 01VSF16007; German Clinical Trials Registration: DRKS00014748). The APVEL study aimed to evaluate specialised PHC in relation to general PHC in North Rhine, Germany.

Due to the complexity of the subject and the wish to develop a ‘more comprehensive view’ [17], data collection was performed in a mixed methods design by conducting a focus group in March 2018 and a postal survey in July/August 2018 in an exploratory sequential design [17]. The exploratory sequential design means to explore a research topic by using qualitative methods to design a quantitative measuring instrument, like a questionnaire, and perform quantitative data collection with this instrument afterwards [17]. The focus group aimed to gain a deeper insight into the topic and develop a questionnaire based on this. The survey aimed to generalise the qualitative results [17].

### Ethics

The ethical review committee of the Medical Faculty of the University of Cologne approved the study (vote number 17-297).

## Data collection

### Inclusion criterion

For data collection the following inclusion criterion was applied: working as a GP in a medical practice in North Rhine.

### Focus group

**Figure 1.**
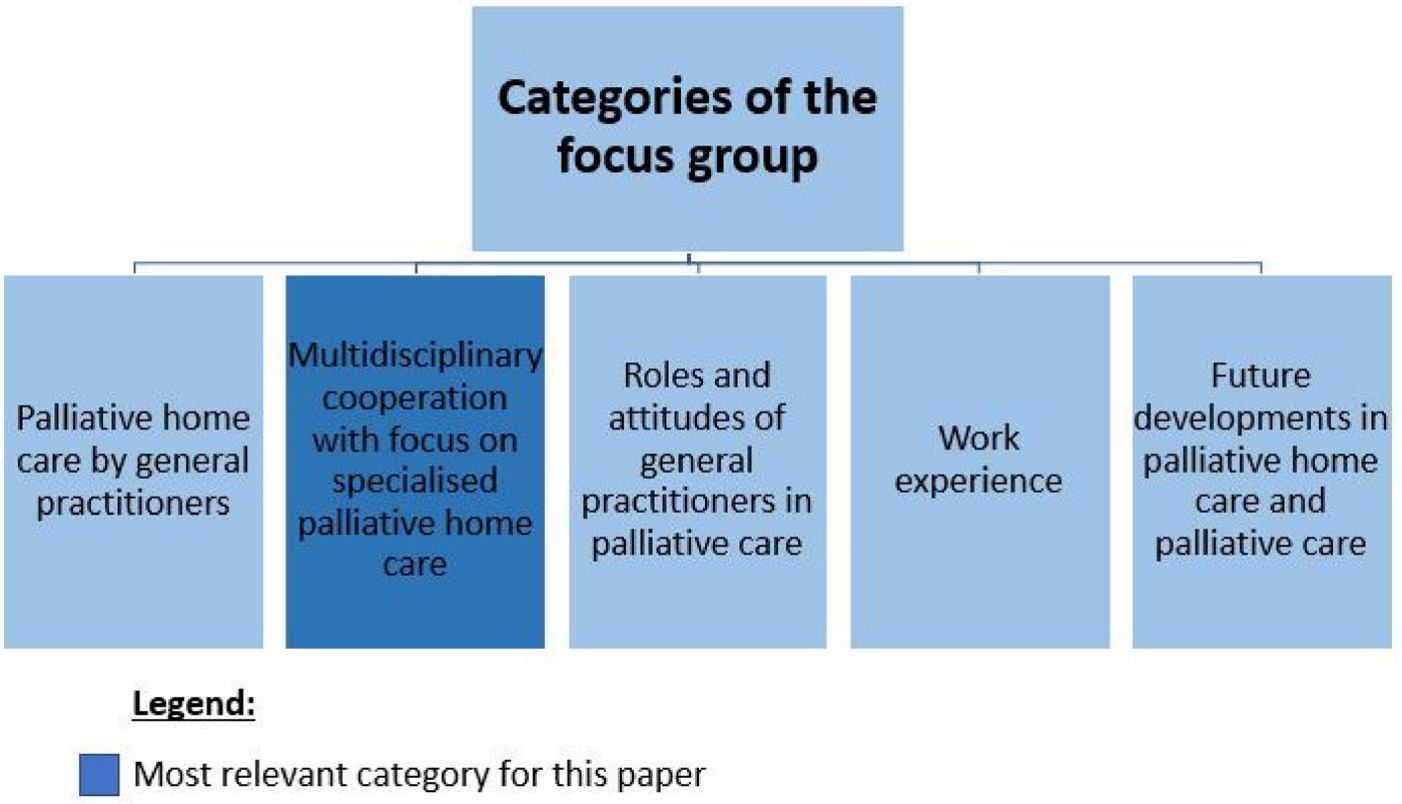
Categories of the focus group.

The researchers started by conducting a semi-structured focus group discussion (categories shown in Figure 1). The interview guide and the deductive part of category system were based on a literature search and three interviews with regional PC experts. To recruit participants for the focus group the authors contacted medical teaching practices of the universities of Cologne, Aachen and Bonn (*n* = 509) by fax and leaflets. The participants did not know each other prior to the focus group. It was carried out at the Institute of Medical Sociology, Health Services Research, and Rehabilitation Science of the University of Cologne. In the beginning, there was a welcoming and an introduction round. To get into the topic all participants had to state in one sentence what experience they have with PC. The discussion started directly afterwards. The course of the focus group was oriented towards the category system (Figure 1). The focus group lasted 94 min, was audio-recorded and transcribed verbatim.

SP reviewed the transcript after finalisation to avoid misunderstandings. SP performed moderation of the focus group, SN and AMV were co-moderators. All three wrote field notes. The notes were used to describe the environment of the focus group. Participants completed a sociodemographic questionnaire (Table 1) and agreed on the study’s modalities by signing a consent form.

**Table 1.** Sociodemographics of participants.

	Focus group *N*	Postal survey *N* (%)
Numbers of participating GPs	6	445**
Gender		
Male	6	217 (48.8%)
Female	–	226 (50.8%)
Missing values	–	2 (0.5%)
Age in years (Mean, age range)	57 (minimum: 52; maximum: 62)	53.5 (minimum: 33; maximum: 78)
Highest PC qualification		
None	2	198 (44.7%)
3 months’ work experience in an inpatient PC facility	–	21 (4.7%)
Basic PC qualification*	1	162 (36.6%)
BQKPMV*	–	10 (2.3%)
Advanced PC qualification*	3	52 (11.3%)
Working experience in years (Mean)	27	18.1
Practice owner	6	364 (83.1%)

*Basic PC qualification: 40 h PC training course (PC basic course).

BQKPMV: qualification in ‘specially qualified coordinated palliative medical care’.

Advanced PC qualification: being a PC specialist with 120 h PC training course (qualified PC physician).

**In analysis, 18 GPs were excluded due to currently working in a specialised PHC team.

### Postal survey

**Figure 2.**
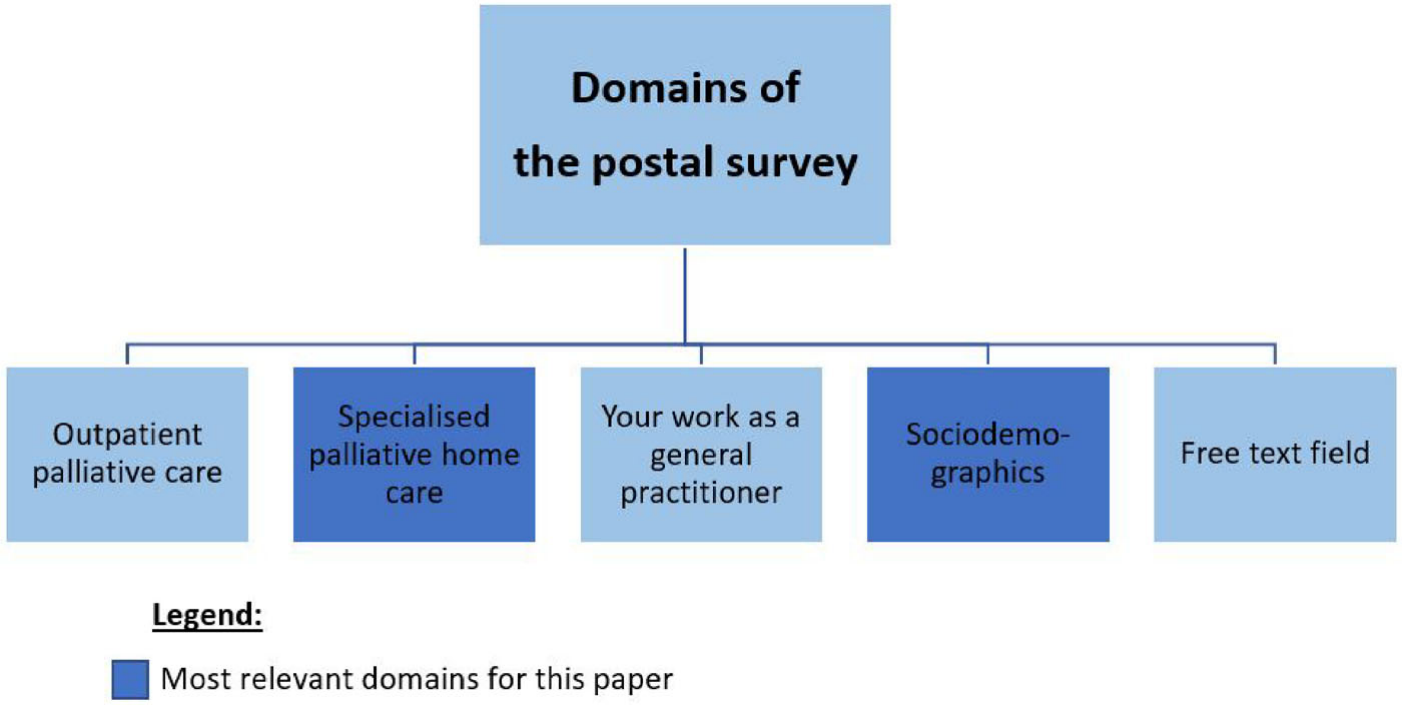
Domains of the survey.

Building on all qualitative findings the authors generated a survey. The questionnaire included 109 items divided into 4 domains (Figure 2). The questionnaire includes both open and closed questions and a free text field. The items of the domain ‘specialised PHC’ were mostly self-developed based on the focus group. For this purpose, the authors extracted the most highly emphasised factors influencing specialised PHC from the focus group data and discussed the findings compared to literature and previous expert interviews. After the discussion, SP and AMV formulated the key points (e.g. degree of involvement, barriers and facilitators of involvement) as questions and put them into a questionnaire format.

Some items are parts of an already existing, non-validated scale [18]. These items are marked with literature references in the following.

The participants in the focus group pretested the questionnaire according to readability, comprehensibility and whether the questions are answerable. The participants could also state whether they are still missing questions in the questionnaire in terms of content. The wording was changed in a few places but no new content was added. Afterwards, the survey was sent by mail to all GPs who cooperate with statutory health insurance funds (*n* = 2199). We have obtained the addresses from a central register that includes North Rhine’s physicians. Data collection was anonymous. According to Dillman’s Total Design Method [19] the researchers invited participants to answer the questionnaire on three occasions.

### Questions derived from the qualitative outcomes

Based on the focus group results, the involvement of GPs has been identified as a key issue. The researchers derived the following questions for the survey:

Realised and desired involvement of GPs in specialised PHC: Do GPs wish to stay involved in specialised PHC patients’ care? And is there a match between the realised and desired involvement of GPs in specialised PHC?

Does realised involvement in specialised PHC correlate with satisfaction with specialised PHC teams?

### Definition of key concepts: (realised and desired) involvement

Involvement as a central part of the research questions was defined as part of PC provision. Involvement means at least being informed about PC provision by other health services providers or mutually coordinating medical treatments as in case conferences or consultations. The most potent form of involvement is to perform medical services even after referral to specialised PHC. Realised involvement means that GPs assumed tasks in specialised PHC patients’ care. Desired involvement is GPs’ hoped-for cooperation with specialised PHC teams: GPs were asked whether they want to stay involved in their patients’ treatment, be informed about the treatment or provide medical services themselves after a referral to specialised PHC.

### Data analysis

#### Qualitative analysis of the focus group’s data

Concerning the focus group’s data, SP and AMV performed a content analysis iteratively and independently using inductive and deductive categorisation in MAXQDA 12. Data analysis started with deductive, literature-based categories. In the next step, inductive categories were derived from the participants’ statements. After three coding runs per coder, SP and AMV discussed their results and developed a finalised category system. SP carried out the final coding. Results were discussed in the research group (AMV, SN, SP) and with the participants by presenting a summary of the results [20]. The researchers agreed that after the focus group the qualitative research goal was reached: the authors had an overview of the current PC situation for GPs in North Rhine and could start developing a questionnaire on this basis.

#### Quantitative analysis of the postal survey’s data

Statistical analysis was performed by using descriptive and regression analysis in Stata 15. We calculated different models to examine the correlation between the desire for involvement and satisfaction with cooperation. To explore the correlation with the wish to be informed, involved or provide medical services after referral to specialised PHC (4-point scales, 1: completely disagree, 4: completely agree), we calculated three different models (see Table 2). In addition, we calculated a model to identify correlations with satisfaction regarding cooperation with the specialised PHC team (4-point scale, 1: unsatisfied, 4: satisfied, see Table 3). Due to the continuously scaled dependent variables, we calculated linear regression models.

**Table 2.** Factors correlating with the desire for general practitioners’ involvement in specialised palliative home care (linear regressions).

		After specialised palliative home care referral I would like to…		
		Stay informed about patients’ care Coefficient (*p*-value)	Stay involved in patients’ care Coefficient (*p*-value)	Provide medical services myself Coefficient (*p*-value)
Qualification	None (reference)			
	3 months work experience in an inpatient palliative care facilities	−0.035	0.010	0.209
		(0.838)	(0.574)	(0.293)
	Basic palliative care qualification°	0.026	0.063	0.144
		(0.748)	(0.461)	(0.136)
	Specially qualified coordinated palliative medical care (BQKPMV)°	0.098	−0.016	−0.151
		(0.671)	(0.948)	(0.575)
	Advanced palliative care qualification °	0.147	0.025	0.170
		(0.276)	(0.856)	(0.279)
Satisfaction with the cooperation with specialised palliative home care teams [18]		−0.054	−0.073	−0.181
		(0.276)	(0.162)	(0.002)
‘Palliative care is general practitioners’ duty’		0.108	0.445	0.388
		(0.145)	(0.000)	(0.000)
Gender (male (reference))		0.021	0.103	0.040
		(0.776)	(0.188)	(0.650)
Work experience in years		0.004	0.002	0.006
		(0.379)	(0.660)	(0.223)
Working as an employee (no (reference))		0.066	0.113	−0.010
		(0.545)	(0.321)	(0.936)
Constant		2.962	1.467	1.695
		(0.000)	(0.000)	(0.000)
*N*		382	380	377
Missing values		39	38	40
*R* ^2^		0.020	0.112	0.117

Legend.

Basic PC qualification: 40 h palliative care training course (palliative care basic course).

BQKPMV: is a qualification in ‘specially qualified coordinated palliative medical care’.

Advanced PC qualification: being a palliative care specialist with 120 h palliative care training course (qualified palliative care physician).

**Table 3.** General practitioners’ satisfaction with specialised palliative home care teams (linear regression).

General practitioners’ satisfaction with specialised palliative home care teams		Coefficient	Standard error	*p*-value	95% confidence interval	
(Un)matching desired and realised involvement in specialised palliative home care	Unmatching – involvement less than desired (reference)					
	Unmatching – involvement beyond desire	0.490	0.184	0.008	0.128	0.853
	Matching	0.514	0.075	0.000	0.366	0.661
Qualification	None (reference)					
	3 months of work in an inpatient palliative care facility	−0.405	0.179	0.024	−0.756	−0.053
	Basic palliative care qualification^a^	−0.178	0.083	0.033	−0.341	−0.015
	Specially qualified coordinated palliative medical care (BQKPMV)^a^	−0.472	0.245	0.055	−0.955	0.011
	Advanced palliative care qualification^a^	−0.186	0.132	0.160	−0.446	0.074
‘Palliative care is general practitioners’ duty’		0.041	0.079	0.602	−0.114	0.196
Work experience		−0.010	0.004	0.025	−0.018	−0.001
Gender		0.061	0.075	0.420	−0.087	0.209
Being an employee (no (reference))		0.035	0.117	0.767	−0.196	0.266
Constant		3.30	0.361	0.000	2.587	4.008
*N* = 346; *R*²=0.173; own calculations.						

^a^Basic PC qualification: 40h palliative care training course (palliative care basic course); BQKPMV: is a qualification in ‘specially qualified coordinated palliative medical care’; Advanced PC qualification: being a palliative care specialist with 120h palliative care training course (qualified palliative care physician).

## Results

### Study population

Sociodemographic data are shown below (Table 1). The focus group included seven physicians (six GPs, one oncologist). 2154 GPs were contacted for the survey (response rate: 20.6%). Eighteen GPs were excluded from the analyses as they worked for a specialised PHC team.

### Outcomes of the focus group

The focus group described the cooperation between GPs and specialised PHC as good and getting even better. Cooperation between GPs and PC specialists was mainly favourable for GPs when specialised PHC makes GPs’ work more accessible. An advantage of cooperation between GPs and specialised PHC teams is the knowledge of GPs concerning their patients from which the PC specialists may benefit. However, GPs’ satisfaction with specialised PHC teams’ work varies. One GP cited: ‘There were specialised PHC teams who were know-it-alls. Of course, that’s not a good way [to cooperate]’ (P02). The degree of desired or realised involvement in specialised PHC may vary: some GPs (want to) stay informed about their patients’ specialised PHC treatment, while others are actively involved or continue to provide medical services themselves. We used this definition of different degrees of involvement described in the focus group as the basis for developing a scale for the survey (s. Outcomes of the survey, Realised and desired specialised PHC involvement).

The participants identified some barriers and facilitators to involvement and cooperation in specialised PHC: As a possible barrier, specialised PHC physicians’ and GPs’ attitudes and personality traits were named. Also, GPs role perception, lack of willingness to cooperate and knowledge gaps about specialised PHC may influence cooperation and involvement negatively. Additionally, communication between GPs and specialised PHC teams is lacking. In contrast, mutual consultations of GPs and specialised PHC teams were named as factors promoting involvement. ‘Taking everybody on board’ (P06), including GPs, is rated positive for specialised PHC’s quality.

### Outcomes of the survey

#### Realised and desired specialised PHC involvement

**Figure 3.**
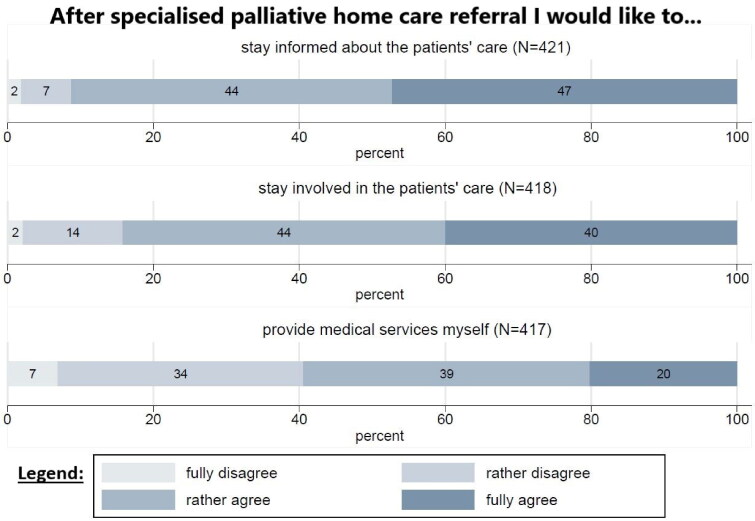
GPs’ desired involvement in specialised PHC patient’s care.

Most participants completely agree (47.3%) or rather agree (43.9%) that they wish to stay informed about their patients’ specialised PHC treatment. Most GPs completely (40%) or rather agree (44.3%) that they want to stay involved in their patients’ treatment after specialised PHC referral. One-fifth of the GPs completely and 39.3% rather agree that they wish to provide medical services themselves after referring patients to specialised PHC (Figure 3).

Comparing the realised and desired involvement, 52.6% of GPs experience a match of desired and realised involvement in specialised PHC, while 42.8% are less involved and 4.6% are more often engaged than expected.

Regarding possible factors correlating with the desire for involvement after specialised PHC referral, linear regressions show the following significant effects: The attitude that PC is GPs’ duty correlates significantly with the desire for involvement in specialised PHC (coefficient: 0.445) or provide medical services (coefficient: 0.388). The desire to provide medical services is significantly correlated with dissatisfaction with the cooperation with the specialised PHC team. There is no statistically significant correlation between desired involvement and GPs’ qualification, gender or work experience in the multivariate analysis (Table 2).

### Does realised involvement in specialised PHC correlate with GPs’ satisfaction with specialised PHC teams?

**Figure 4.**
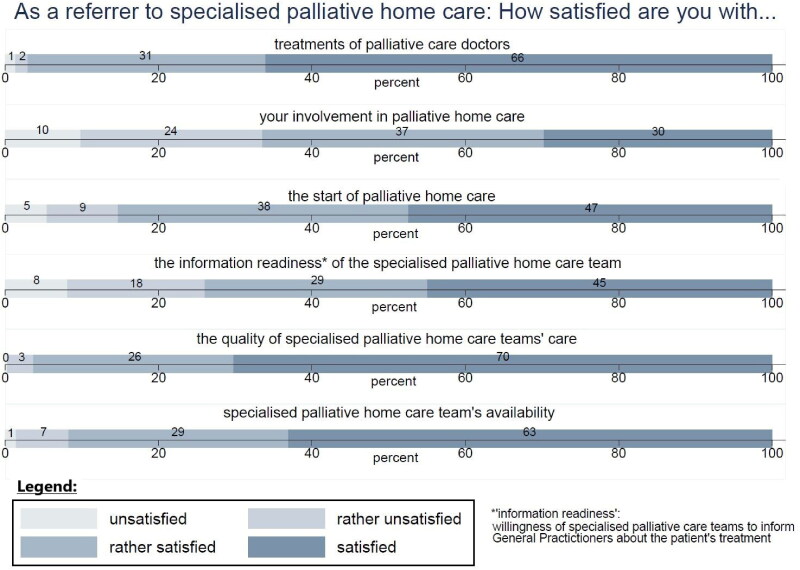
GPs’ satisfaction as an specialised PHC referrer (oriented on KEF-CH [18]).

Asked about their satisfaction as specialised PHC referrer, GPs were most satisfied with the quality of specialised PHC teams’ care (Mean: 3.7; 1: unsatisfied, 4: satisfied), treatment by PC physicians (Mean: 3.6) and specialised PHC teams’ availability (Mean: 3.5) (Figure 4).

In the linear regression model (Table 3, *n* = 346, 81 GPs were excluded from the model due to missing data), significant positive associations were found between satisfaction regarding cooperation with the specialised PHC team and matching as well as involvement beyond that desire. Basic PC training, 3 months of work in an inpatient PC facility and work experience, significantly negatively affected satisfaction with specialised PHC teams.

## Discussion

### Main findings

Our study shows that GPs are primarily satisfied with specialised PHC teams’ care, although they reported in the focus group that cooperation is not always optimal. However, GPs were less satisfied with specialised PHC when their PC knowledge was higher. GPs are often less involved in specialised PHC than they would like, especially when they have higher PC qualifications.

The postal survey shows that most GPs wish to stay informed and be (actively) involved after referring to specialised PHC. According to realised and desired involvement in specialised PHC, 42.8% of the GPs who participated in the study are less involved than desired, which supports the focus group’s data of varying satisfaction. In addition, realised involvement of GPs positively correlates with satisfaction with specialised PHC teams if it matches or is beyond their desired involvement. Nevertheless, basic PC training, as well as 3 months of work in an inpatient PC facility and work experience show a negative correlation on satisfaction with specialised PHC teams.

### Comparison with existing literature

Our findings show mostly good cooperation of GPs and specialised PHC. Similar results were found by Koper et al.: In their study, Dutch GPs describe positive experiences with involvement in PC [4]. Per our finding that satisfaction is variable, other studies also found that collaboration in PHC can be challenging: Senior et al. and Keane et al. describe tensions between generalists as GPs and PC specialists [3,21]. GPs may feel ‘deskilled’ by PC specialists [3]. Gardiner et al.[22] refer to this phenomenon as ‘professional territorialism’ while Kaiser et al. encourage the overcoming of competition between PC specialists and GPs [23].

We found in our data a high satisfaction of GPs involved in specialised PHC more than they desired. This is in accordance with the findings that GPs see themselves as important PC providers [14]. The participating GPs’ satisfaction of staying involved in specialised PHC could be interpreted as a confirmation that they see themselves as significant PC providers even after specialised teams are involved.

### Strengths and limitations

The research team included broad expertise in qualitative and quantitative research. Some benefits of the mixed methods design lie in compensating the methodological weakness of individual methods (e.g. lower significance of focus group results due to few participants; self-selection bias; lack of detailed explanations for survey results). However, there are some limitations: the study relies on a regional data collection, although North Rhine is well suited as a German example region having the average number of specialised PHC referrals [24]. Data was collected in 2018. The response rate of the survey is average compared to other German studies with GPs supported by sending reminders [19,25]. In international comparison response rates of German GPs are generally low [25]. The participating GPs were more knowledgeable about PC than the average GP. In this study, 11.3% of the participants had an advanced PC qualification, while the average of outpatient physicians with this qualification is 3.9% [26]. This disproportion reduces the representativeness of the data. It would have been interesting if more GPs without training in PC had participated in the survey. We assume that GPs particularly interested in PC participated in our survey. A special incentive for a higher participation rate of less interested or less PC-trained GPs could have helped here. In addition, more emphasis could have been placed on the benefits of the study for the daily working practice of GPs to address their ‘contribution to the common good’ as a reason for participation [25]. The age and gender of the participants are about average [27,28].

Reasons for suboptimal involvement of GPs in specialised PHC cannot be derived in more detail from our data. There can be diverse reasons, which can be found on both sides: the GPs (based on lack of PC knowledge, lack of time to provide PHC) or the specialised PHC teams (leaving GPs out of the PHC) [14]. GPs who are part of a specialised PHC team were excluded from analysis because of a potential bias based on competing interests.

The *R*^2^ for the regression models are low indicating that the variables included in the model only partially explain involvement and satisfaction. Hence, there are other undiscovered determinants as well, which should be identified through further research.

The following procedures could also have positively supported the data quality: Clustering the survey’s data on level of the specialised PHC teams (not possible for data protection reasons); conducting further focus groups to strengthen the reliability and add more explanatory variables to the analysis (not possible due to low willingness of physicians to participate in the focus group: only six GPs out of 509 practices answered our request to participate. However, the focus group had a sufficient number of participants [29]); extending the survey’s pre-test non-focus group participants (not possible due to the problems in recruitment).

The study’s description orientates on a reporting checklist and uses statistical (Stata 15) as well as qualitative data analysis software (MAXQDA 12) [16].

### Implications

Strong working relationships between specialised PHC teams and GPs must be established to improve collegial exchange and close the gap between GPs’ desired and realised involvement. Structural conditions need to be created for this, for example, by providing incentives for communication between GPs and specialised PHC teams.

For this, the following three steps for specialised PHC teams are essential for better involvement of GPs regardless of their training level: (1) inform GPs about patients’ care after the referral is made, (2) involve GPs in PC patients’ treatments if possible and feasible, and (3) allow GPs’ provision of their medical services to the jointly treated PC patients.

## Conclusion

GPs appreciate specialised PHC and its provision. Cooperation between GPs and specialised PHC teams is seen mostly positive by GPs with the potential to improve. Also, the integration of GPs varies and should be more balanced to close the gap between GPs’ desired and realised involvement in specialised PHC. This could increase GPs’ satisfaction with specialised PHC because GPs would not have to feel that they let their patients down by (being forced to) hand over the treatment entirely to the specialised PHC team.

## Appendix 1. Good reporting of A mixed methods study (GRAMMS)

(O’Cathain A et al. [16])

**Table:**

(1)	Describe the justification for using a mixed methods approach to the research question – see study design
(2)	Describe the design in terms of the purpose, priority and sequence of methods – see study design
(3)	Describe each method in terms of sampling, data collection and analysis – see data collection
(4)	Describe where integration has occurred, how it has occurred and who has participated in it – see data collection & results
(5)	Describe any limitation of one method associated with the present of the other method – see discussion, strengths and limitations
(6)	Describe any insights gained from mixing or integrating methods – see discussion, main findings

## List of abbreviations

APVEL: Evaluation of specialised palliative home care in North Rhine (German: **E**va**l**uation der Wirksamkeit von S**APV** in Nordrhein)
PC: Palliative care
PHC: Palliative home care
